# AI-Powered Drug Classification and Indication Mapping for Pharmacoepidemiologic Studies: Prompt Development and Validation

**DOI:** 10.2196/65481

**Published:** 2025-06-12

**Authors:** Benjamin Ogorek, Thomas Rhoads, Eric Finkelman, Isaac R Rodriguez-Chavez

**Affiliations:** 1 Spencer Health Solutions, Inc Morrisville, NC United States; 2 4Biosolutions Consulting Rockville, MD United States

**Keywords:** generative language model, artificial intelligence, AI, large language models, LLMs, natural language processing, NLP, drug classification, Anatomical Therapeutic Chemical, ATC, spencer device, smart hub

## Abstract

**Background:**

Pharmacoepidemiologic studies, which promote rational drug use and improve health outcomes, often require Anatomical Therapeutic Chemical Classification System (ATC) drug classification within real-world data (RWD) sources. Existing classification tools are expensive, brittle, or have restrictive terms of service, and lack context that may inform classification itself.

**Objective:**

This study sought to establish large language models (LLMs) as an assisting technology in the drug classification task. This included developing artificial intelligence prompts that reason about drugs using RWD and showing that the resulting accuracy, efficiency, and effectiveness are favorable to alternative methods.

**Methods:**

A prompt was constructed to classify aspirin as either an analgesic or antithrombotic and evaluated within 12,294 anonymized daily dose strings from a polychronic population residing in the United States and Canada. The patients used a smart medication dispenser called “spencer” and consented to the use of their data for research. The LLM prompt requested that the best and next-best second-level ATC code be returned, and grading was performed on a 3-point scale. After success in a pilot sample of 20, an inference sample of 200 was taken without replacement. Finite population inference was carried out on the proportion of outputs receiving 1 of the top 2 grades. As a benchmark, Google’s Programmable Search Engine was used to query the drug name plus “ATC code” followed by regex-based extraction of ATC codes. All imperfect results were reviewed.

**Results:**

The population consisted of 12,294 daily dose strings from 86.26% (2908/3371) patients residing in Canada and 13.73% (463/3371) residing in the United States. A prompt using the chain-of-thought reasoning was able to distinguish between aspirin’s analgesic versus antithrombotic therapeutic uses and performed well in the pilot sample. In the inferential sample, 87.5% (175/200) were graded as perfect, 5% (10/200) had a minor issue, and 7.5% (15/200) had a major issue. The estimate of the proportion of at least mostly correct classification was 92.5% (185/200, 80% CI 90.1%-94.9%). For the search-based algorithm, 82.5% (165/200) were deemed acceptable. The chain-of-thought reasoning was most helpful with supplements (eg, folic acid) when high doses indicated antianemic preparations. The problem formulation of daily dose inputs and multiple ATC outputs was sometimes incompatible with the drug (eg, pregabalin, calcitriol, and methotrexate).

**Conclusions:**

GPT-4o offers cost-effective drug classification from RWD without violating any terms of service. Using a chain-of-thought prompting technique, GPT-4o can reason about drug dosages that affect the class. The wide accessibility of LLMs gives every research team the ability to classify drugs at scale, a key prerequisite of pharmacoepidemiologic research.

## Introduction

### Background

Pharmacoepidemiology is a “bridge science” between epidemiology and clinical pharmacology that has, since its formative years, encompassed wide-ranging fields such as epidemiology, pharmacology, medicine, biostatistics, and social sciences [1]. It is positioned to benefit from advances in an even wider array of disciplines, including bioinformatics, data science, machine learning, artificial intelligence (AI), natural language processing, large language models (LLMs), systems pharmacology, pharmacogenomics, pharmacometabolomics, and health informatics [2-7]. These advances are integrated into pharmacoepidemiologic research to discover ineffective or unsafe drugs, encourage better prescription decisions, and reduce unnecessary health care costs [8].

LLMs are assistive tools that process broad ranges of factual knowledge, opening new avenues for synthesizing information across diverse fields. Their incorporation into pharmacoepidemiology holds great potential for redefining how we can understand the safety, efficacy, and use patterns of prescribed medications in large patient populations and reduce unnecessary costs.

### Pharmacoepidemiology and the Anatomical Therapeutic Chemical Classification System

Pharmacoepidemiologic research depends on a concept of *drug class*, a classification of drugs regarding mechanism of action, therapeutic intent, or chemical structure, with the Anatomical Therapeutic Chemical Classification System (ATC) classification system considered mandatory [8-11]. Applications of the ATC methodology according to the World Health Organization are included in Table 1.

**Table 1:** Applications of the Anatomical Therapeutic Chemical Classification System (ATC) methodology according to the World Health Organization.

Application	How ATC codes are used
National standard for medicinal products	Used as a national standard for the classification of medicinal products for various countries
International classification	Provides a common language for describing drugs
Health policy	Drug use statistics using ATC codes are used to improve the quality of drug use in a population
Pharmacoepidemiology or drug use research	To study trends and patterns in drug use
Pharmacovigilance	For linking adverse drug reactions to ATC classes
Assisting procurement agencies and payer organizations	To gain an overview of the availability of drugs and reduce the risk of drug shortages

The ATC system classifies active substances (ie, drugs), including combinations of active ingredients, into groups at 5 different levels. This 5-level structure of the ATC system enables a hierarchical classification, which supplies broad and specific categorization of drugs, and helps in different levels of analysis in pharmacoepidemiologic studies. Using the example of lisinopril and hydrochlorothiazide, the first level ATC code is “C” for cardiovascular, the organ or system acted upon, the second-level ATC code is “C09” for agents acting on the renin–angiotensin system, and the fifth-level ATC code is “C09BA03”: lisinopril and diuretics [12,13]. While there is the basic principle of 1 ATC code for each drug [12], here the fifth-level ATC code is still ambiguous due to the presence of an unspecified diuretic.

The ATC system is not suitable for guiding decisions about reimbursement, pricing, and therapeutic substitution [14], and thus, real-world data (RWD) typically lack ATC codes, rather using drug codes that support day-to-day operation and logistical functions within health care and pharmaceutical systems. This discrepancy between research needs and RWD structures presents a significant challenge for pharmacoepidemiologists.

In the United States, most pharmacy management systems and electronic health record systems use National Drug Codes (NDCs), which were designed for inventory management and reimbursement and do not directly identify a drug class [9,15]. In Canada, the analogous system is the Drug Identification Number (DIN) [16]. In the Netherlands, it is the Z-Index [17]. To conduct pharmacoepidemiologic research with these data sources, accurate ATC classification of drugs becomes an essential task to the research process. This necessity for accurate drug classification underscores the potential value of advanced computational methods, such as machine learning and natural language processing, in bridging the gap between diverse drug coding systems and the standardized ATC classification required for robust pharmacoepidemiologic analysis.

### Tools to Map Prescribed Drugs to ATC Classes

The Center’s ATC/defined daily dose Index web utility includes a query engine for drug name strings [13]. While there is no published application programming interface (API) for the search tool, adding /?name=<drug> to the end of the URL will execute the search and could set up a web scraping task. Fuzzy matching is limited to the options of “containing query” and “starting with query,” which is restrictive in practice. For example, in either case, the drug string “apo-domperidone” returns no results while “domperidone” returns 3.

ATC codes appear in Wikipedia infoboxes which suggest the use of DBpedia [18]. Querying DBpedia, however, requires exact resource names, for instance, “dbr:Metformin.” The DBpedia organization does provide a lookup service [19] for text queries, like the ATC/defined daily dose Index, but it is also thrown off by variations in the drug’s name.

When operational drug codes (eg, NDC or DIN) are in the database, a different suite of tools becomes available. In the United States, the National Library of Medicine offers a service around RxNorm, a standardized nomenclature for clinical drugs [20], which links NDC to fourth-level ATC code [21]. Health Canada’s Drug Product Database [22] allows joining on the DIN directly. For countries with languages spoken by fewer people, the task is more difficult. Researchers in the Netherlands, for instance, had to resort to multiple data sources and extensive data cleaning to build their Dutch ATC ontology, and it still requires manual tasks [23].

DrugBank offers a full-text search built on top of Amazon Web Services Elasticsearch that can map drug names in various forms to standard terminology [24,25]. A DrugBank search result includes the full list of appropriate ATC codes, synonyms, indications, and more. While DrugBank is offered to the public and academia for free, commercial use requires a paid license [26]. The reliance on such commercial products can limit the accessibility of crucial drug classification tools, potentially creating disparities in research capabilities between well-funded and resource-limited institutions.

A commercial option that is inexpensive, yet controversial, is the use of Google’s Programmable Search Engine via the Custom Search JavaScript Object Notation (JSON) API [27]. The results contain the data essential to displaying search results on a website, and while they do not include the entire content of each site, they contain a “snippet” which often includes key information of interest. While recent US court decisions indicate a trend toward more leniency in web scraping of publicly accessible data, such as hiQ Labs versus LinkedIn [28], Google’s terms of service explicitly prohibit storing information obtained from the service in “any nontransitory manner” [29]. This, in principle, makes possible a claim against the user for breach of contract, but the actual enforceability of these terms is questionable [30].

### ATC Drug Classification Challenges in a Multiregional Smart Medication Adherence System

Integration into the medication database of the ATC classification is critical to ensuring robust pharmacoepidemiologic research across a wide array of health care systems. This section demonstrates the challenges and limitations of the implementation of the ATC classification in the context of a real-world smart medication dispenser.

**Figure 1:**
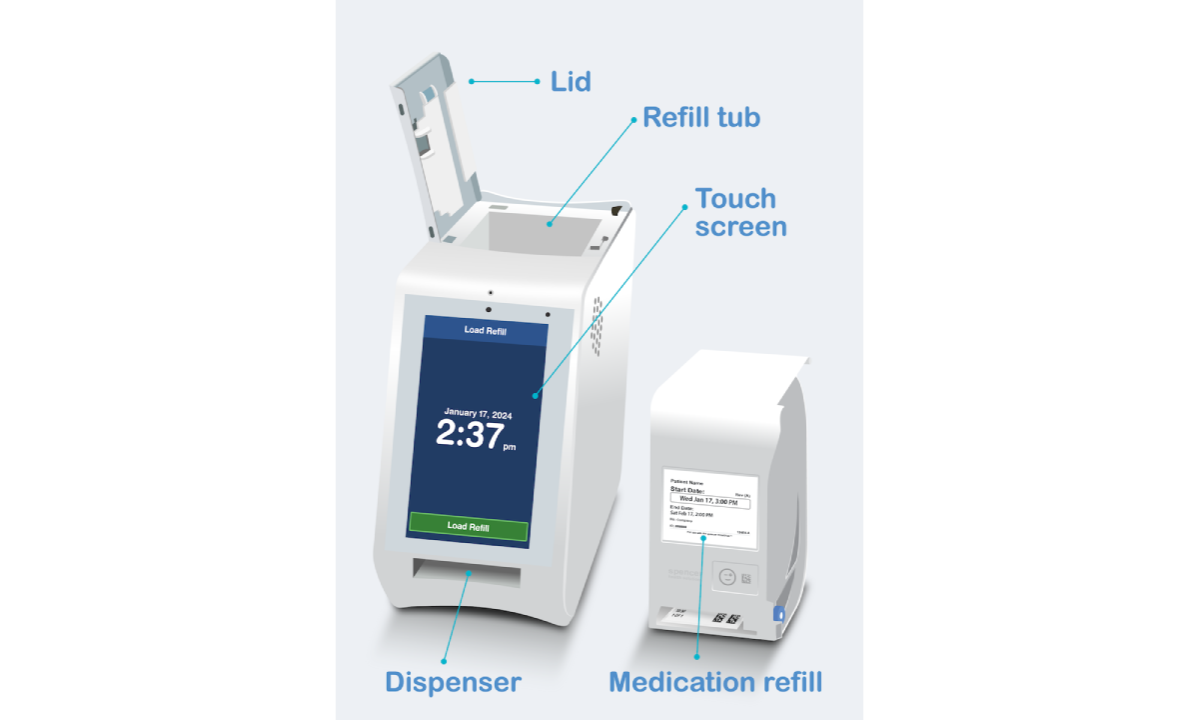
Key components of the spencer smart medication dispenser.

Spencer Health Solutions (SHS) is a health technology startup that manufactures “spencer” [31], a smart medication dispenser that dispenses strip-packaged oral solid tablets (Figure 1). At the time of drug refill creation, records are inserted into a database (maintained by SHS), which includes the scheduled time of each pouch and information on the drugs it contains. A separate set of drug records contains drug name, drug code (NDC for US drugs, DIN for Canadian drugs, and Z-Index for Dutch drugs), and strength description.

The spencer device shows the potential of smart medication dispensers to generate RWD that are useful in pharmacoepidemiologic studies, but there have been difficulties with relying on operational drug codes to obtain second-level ATC codes. First, many of the drugs in the SHS drug database do not contain valid NDC or DIN codes, especially supplements such as *advanced 4-strain probiotic* or *webber naturals womens 50 plus most* (as they are abbreviated in the database). In addition, when the daily dose informs the therapeutic subgroup, NDC and DIN codes are insufficient, since patients often take multiple pills per day. For example, aspirin at low daily doses (75-100 mg) is a match to second-level ATC code B01 (antithrombotic agents), whereas aspirin, whereas aspirin at doses >325 mg maps to N02 (analgesics, a second-level ATC code), that is, analgesics. Finally, since the original drug classification exercise, patients began using spencer in the Netherlands, with Z-index values populating the drug code field in the European database. Although out of the scope of this paper, the existing classification pipeline based on 2 region-specific methodologies is unhelpful for this new drug source. These challenges indicate both the complexity of drug classification in multiregional systems, and that more sophisticated, context-aware approaches would be required, accounting for differences in dosage, formulation, and regional coding systems.

The limitations seen in the real-world application set a clear case for the need for a more flexible, comprehensive, and automated approach to drug classification. Advanced computational methods, such as machine learning, natural language processing, and AI, can help address these challenges by providing more accurate, adaptable classification systems dealing with diversified drug information across different regions and contexts of health care.

### Drug Classification Using GPT-4o

In recent years, LLMs have shown the ability to perform a broad array of tasks with minimal task-specific training using textual prompts with “few-shot” and “chain-of-thought” prompting techniques [7], enabling the automation of tasks that are currently difficult and manual. GPT-4o [32], OpenAI’s most advanced model at the time of writing the initial manuscript, contains a wealth of information about pharmaceutical drugs as well as the ATC classification system. In interactive exploration, GPT-4o was good at reasoning about second-level ATC level codes from drug inputs that had a variety of input formats. This feature of flexibility in handling various formats of input is valuable as sources of drug information are heterogeneous among different health care systems and across countries. In addition, a GPT-4o prompt can include additional information in an efficient and optimized manner about the daily dose that a patient is taking, which is useful when the dose may influence the final ATC classification.

The application of LLMs to drug classification presents a novel approach that could potentially address many of the limitations of traditional methods of drug classification. In this context, a large pharmacoepidemiologic knowledge base that uses LLMs (eg, GPT-4o) is robust in its handling of regional variations, dose-dependent classification, and data harmonization across different health care systems. Therefore, LLMs may improve multiregional pharmacoepidemiologic research capabilities related to drug classification challenges.

### Objectives

This paper sought to establish LLMs as assisting technology in the drug classification task. This includes developing AI prompts that reason about drugs using RWD and showing that the resulting accuracy, efficiency, and effectiveness are comparable to alternative methods. The developed prompts should be available immediately to classify drugs under a wide range of research budgets.

## Methods

### Patient Population

Drug records of patients were included in this study if they met the criteria described in Textbox 1.

No raw drug record was sent outside the corporate network as part of this research. As will be described in the next section, the data submitted to GPT-4o was an irreversibly anonymized set of daily drug dose strings.

**Inclusion criteria for drug records in the study.**

**Inclusion criteria**

1. The patient resided in the United States and Canada and belonged to a value-based care management organization.

2. The patient signed the care organization’s consent form and agreed to the Spencer Health Solutions’ end-user license agreement, permitting their deidentified data to be used for research purposes.

3. A refill was created with a pouch scheduled to be dispensed on or after January 1, 2024, as queried on June 1, 2024.

### Anonymization Procedure for Daily Drug Doses

This section describes the anonymized extraction of drug dosing data from the database. A drug table in the application database included the drug name, the strength as a text string, and the quantity of pills (including fractions). The drug names were sometimes combinations of a manufacturer and a generic name (eg, “apo-rosuvastatin”) and other times a brand name (eg, “prolopa 50-12.5”). Other times, the drug name would be as generic as “acetaminophen.” Dosage strengths were unstandardized strings such as “50 mg” or “50 MG” and could include multiple active ingredients (“50/200MG” for carbidopa levodopa), a unit of time (“300.0 MG/24HR”), and other variations (while lower case “mg” is more appropriate than upper case “MG” to represent milligrams, the two are used interchangeably in this paper).

To construct a set of inputs suitable for OpenAI’s chat completion API, a string was constructed for each unique drug and daily dose combination, without any other patient information. The drug name was processed by only a lower-case transformation. Drug strength was processed by imputing missing strengths as “UNK” and otherwise left unchanged (strengths of “0” were allowed to pass through). The daily dose processing was described in Textbox 2.

For example, consider a patient prescribed the drug with the name “pms-quetiapine” (generic quetiapine fumarate from manufacturer Pharmascience, Inc) twice per day at 8 AM and 7 PM. At each dispense, 1 pill of strength 200 mg and 2 pills of strength 25 mg are scheduled. Following the steps described in Textbox 2, “pms-quetiapine|2 pills of 200 mg, 4 pills of 25 mg” would be the resulting string input. An example with combination ingredients is 1 pill of “carbidopa/levodopa er” of strength “25/100 MG” scheduled twice daily, or “carbidopa/levodopa er|2 pills of 25/100 MG.” These are anonymized records that cannot be linked back to an individual.

**The processing of the daily dose.**

- Pill quantity was summed by patient ID, date, drug name, and drug strength.
- A pill quantity string was defined within the previous dataset using the formula:
  “{quantity} pill” if quantity=1,
  “{quantity} pills” otherwise
- The previous dataset was aggregated over drug strength within patient, date, and drug name by creating a comma-delimited list of multiple strengths for each drug, taking the form
  “{pill quantity string} of {drug strength}.”
- The patient identifier was discarded, and a distinct operation was performed on the drug name and drug strength string.

### Developing the Classification AI Prompt

The iterative development of the AI classification prompt used a single motivating example, the case of aspirin, and the goal of iterative prompt development was to guide the LLM to classify low doses as ATC code B01 for antithrombotic use and higher doses as ATC code N02 for pain relief. Aspirin is a well-known example of a drug where context matters, and any bias from this specific motivating example will be apparent in the results. The creation of the prompt was an iterative exercise that existed in 2 phases: the initial prompt creation and revision using the pilot sample. In the inference stage, the prompt was fixed, and no further changes were made.

During the initial prompt creation phase, 2 techniques that were available to be used were few-shot learning and chain-of-thought prompting. After achieving a prompt that worked as desired for aspirin at different daily dosing levels, 20 drug names and drug strength strings were randomly sampled from all possibilities observed in the dataset and served as a pilot sample for the initial prompt. These were evaluated by expert review by coauthor IRR-C, with expertise in clinical research, digital medicine, and regulatory affairs.

The pilot sample was sent to GPT-4o via the OpenAI Batch API [33] with all parameters set at their defaults except for the following. Temperature is a parameter that can vary between 0 and 2, where low values (eg, 0.2) result in more consistent outputs, whereas higher values result in “more creative” results (eg, 1) [34]. This research used a temperature of 0, as consistency was a priority. The other nondefault parameter was “max_tokens,” set to 1000, which represents the maximum number of tokens that can be generated in the chat completion [35]. A token is on average three-fourth of a word (100 tokens is about 75 words) [36], and max_tokens was set below the highest allowed value of 4096 to avoid the longest explanations but to still allow for long responses if necessary.

**Figure 2:**
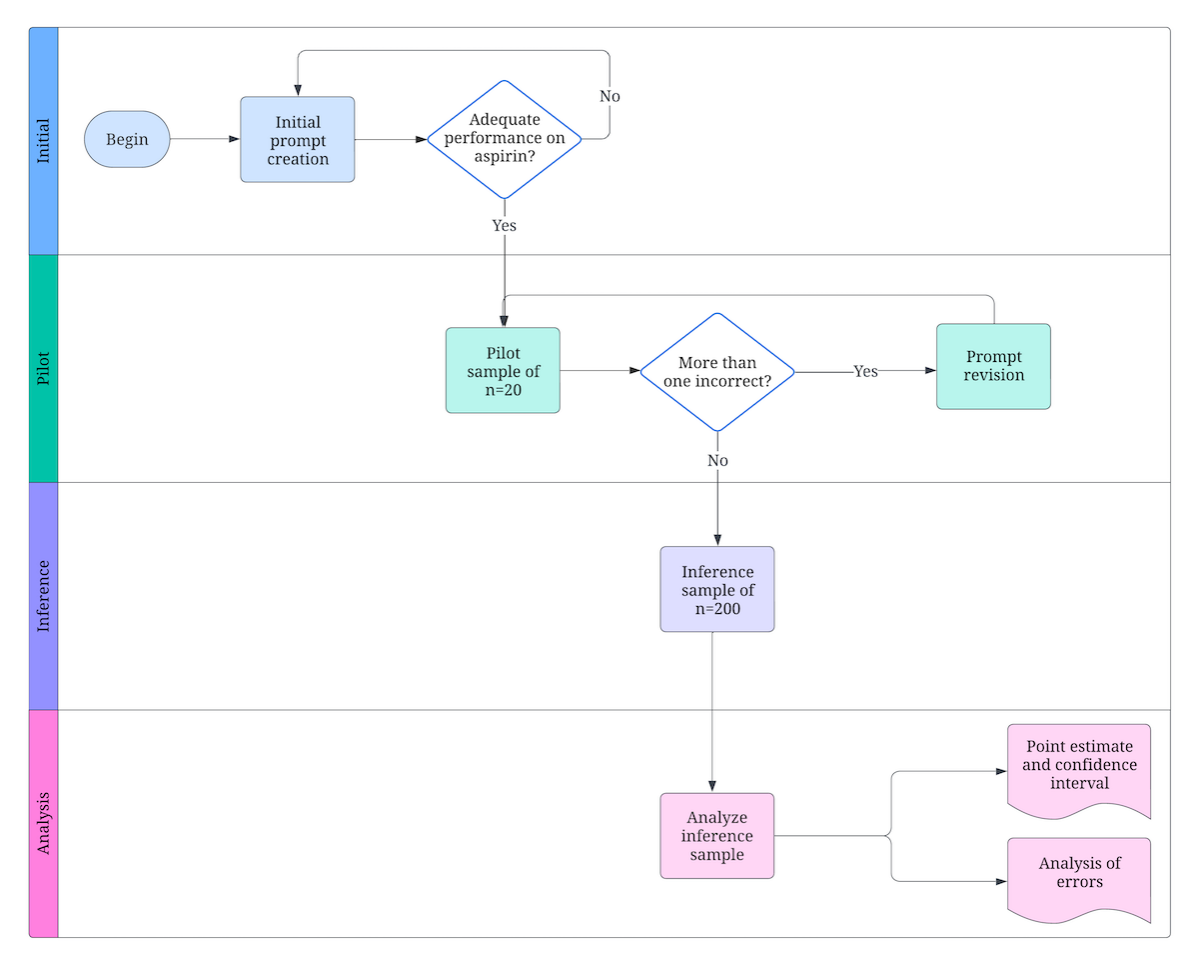
The methodology used to develop and test Anatomical Therapeutic Chemical Classification System (ATC) classification artificial intelligence (AI) prompt.

If more than 1 classification was deemed incorrect, the prompt would be revised before proceeding. The development and validation of this AI-driven classification system were thus optimized through an iterative refinement process guided by expert feedback (Figure 2).

### Finite Population Inference

This section details the statistical methodology that was used in estimating the performance of the GPT-4o model and custom prompt in second-level ATC classification. The methodology focused on the finite population of daily dose strings in the SHS database. Finite population formulas, while not materially different in this case from their infinite population counterparts, were used to emphasize that the inference is made to the specific set of SHS drug prescriptions and not to any larger population. This approach emphasizes the study’s focus on the internal validity of the findings. The finite population sampling approach is model free and has well-understood properties in repeated sampling [37].

The values that were sampled were numerical grades of 1, 2, and 3, generated in the following manner. The output from GPT-4o was graded collaboratively among the authors on a 3-point scale, where a grade of 1 is flawless, a grade of 2 is more correct than incorrect, and 3 is more incorrect than correct. While the counts of each grade will be provided, formal inference to the full population of 12,294 daily dose strings focused on *p*, the proportion of at least mostly correct grades (ie, grades of 1 or 2) in this population. This focused the presentation of performance on a meaningful criterion while reducing inferential complexity.

The finite population sampling estimate of *p* is the sample proportion of outputs that are at least mostly correct in the chosen sample of *n*. The finite population CI is:



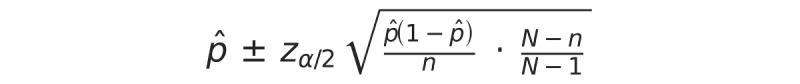



Where N is the number of daily drug dose strings in the full population, n is the sample size, and a is the proportion corresponding to the approximate (1–*a*) 100% CI for population parameter *p*. This formula accounts for the finiteness of the population, giving a better estimate of the CI compared with formulas for infinite populations.

The CI formula was also used to arrive at the sample size. A margin of 0.02 combined with a population proportion of 0.95 and an 80% confidence leads to a sample size of 189, which was rounded up to n=200. The lower level of confidence than the typical 95% was chosen here to lead to a somewhat smaller sample, and there is precedent in making this trade-off in medical research contexts where “reasonable certainty” is acceptable [38].

Each imperfect output (grades of 2 or 3) was examined qualitatively and presented to the reader in tabular form. The errors were then coded into categories, and the frequencies were displayed visually. For mistakes that were difficult to explain, the OpenAI interactive “playground” was consulted for reproducibility.

### The Benchmark of Google’s Programmable Search Engine

When researching a single drug, it is generally easy to find ATC codes by searching for the drug name in Google Search followed by “ATC code.” Despite the issues with the automation of this approach at scale, the drugs from the 200 daily dose strings were sent to Google’s Custom Search JSON API in the form “<drug name>ATC Code.” For each drug, a Python program looped through the top 10 results (the default number of items returned) and searched for strings matching the regular expression “[A-Z]\d{2}[A-Z]{2}\d{2}.” The first 3 characters of these strings were added to a list, and the highest frequency second-level ATC code was the choice made by the search-based algorithm.

The grading of the search-based algorithm was simplified. While the LLM-based algorithm had to find the best and next-best second-level ATC codes, the search-based algorithm only had to find the single best ATC code. There were only 2 grades applied: "appropriate" and "not appropriate," where appropriateness is for a database of oral solids. If there is a tie between 2 second-level ATC codes and both are appropriate, then the output is appropriate. If there is a tie and at least 1 code is inappropriate, then that output is not accepted. This sets up the fraction of appropriate grades as a benchmark, acknowledging that the search-based algorithm is not encouraged to pick ATC codes relating to oral solids.

The results of the search-based algorithm are adjunctive to the results of the LLM, and the decision was made not to pursue formal inference. Comparing the success rates was inevitable, but not the goal of the paper. However, it was informative to see cases where the search-based algorithm failed and the LLM succeeded, and vice versa. A table of cross frequencies was thus compiled, and all cases where the search-based algorithm’s output was graded “not appropriate” were added to the table of errors.

### Ethical Considerations

This study used operational data collected from a commercial medication dispensing system used in routine patient care and was not subject to institutional review board review requirements, so approval was not obtained. Users of the spencer device provided consent for data collection through the End User License Agreement, which covers the collection of medication adherence data and responses to quality of life and patient-reported outcome surveys as part of the system’s standard operation. No additional compensation was provided to users beyond the normal terms of their device use agreement. All data analyzed in this study were deidentified before the analysis. SHS has achieved both ISO 27001 and Data Privacy Framework certifications, and the system uses industry-standard encryption and security measures.

This research analyzed data collected during standard clinical care and device use. All results are presented as anonymous aggregate statistics. The original data collection occurred as part of routine clinical practice, with patients providing consent for research use through the device’s terms of service and care management agreement. Under the Tri-Council Policy Statement: Ethical Conduct for Research Involving Humans, Article 2.4, research ethics board review is not required for research that relies exclusively on the secondary use of anonymous information, where the process does not generate identifiable information [39]. Under US regulation 45 CFR 46 104(d)(4)(ii), institutional review board review is not required when information is recorded by the investigator in such a manner that participants cannot be identified, directly or through identifiers linked to the participants; the investigator does not contact the participants; and the investigator will not reidentify participants [40].

## Results

### The Initial AI Prompt

The initial prompt constructed is provided in Textbox 3 and made available as a plain text file in Multimedia Appendix 1. With a goal of classifying aspirin in a dose-dependent manner with instructions and examples, this prompt requested output consisting of only ATC codes in a pipe-delimited list.

When tested interactively, the initial prompt repeatedly failed to classify high doses of aspirin with N02 as the most likely second-level ATC code. The following inputs all consistently produced the response “B01|N02”: “aspirin|1 pill of 81mg,” “aspirin|2 pills of 325mg,” “aspirin|12 pills of 325mg,” and “aspirin|3 pills of 1000mg.” Sentences were added, such as “Prioritize the dose” and “Think about what conditions the total daily dose would be most likely to treat,” but these were not effective. Results from testing this first AI prompt, with a focus on aspirin classification performance, emphasized challenges faced in the development of a robust classification system that may deal with dose-dependent categorizations. This motivated the creation of a second prompt with chain-of-thought reasoning techniques.

**The initial artificial intelligence (AI) prompt: few-shot learning with concise output.**

# Instructions

You are a classifier of drug prescriptions into Anatomical Therapeutic Chemical (ATC) second level subgroups. As a drug and dose combination may contain multiple ATC second level subgroups, your job is to return to the closest ATC second level category, followed by the next closest. If there is one and only one category, then return “NA” for the second category. If the drug cannot be classified or is ambiguous, return “UNKNOWN” for both ATC fields. Return exactly two fields are separated by a pipe (“|”).

# Input and Output format

- Input: <drug name>|<daily drug dose>

- Output: <Best level 2 ATC Code>|<Next Best level 2 ATC Code (if applicable)>

# Additional Information

All drugs are oral solids. The drug name as a string. Handle different capitalizations, common misspellings, and concatenations with manufacturer names. If the daily drug dose is missing or unintelligible, then do your best with the drug name alone.

# Examples

* Input: metformin|2 pills of 500MG

* Output: A10|NA

* Input: apo-dexamethasone|0.5 pills of 4.0 MG

* Output: H02|S01

* Input: XYZ−1234|1 pill of 1 g

* Output: UNKNOWN|UNKNOWN

* Input: webber naturals womens 50 plus most

* Output: A11|NA

### The Revised AI Prompt Using Chain-of-Thought Reasoning

On the basis the results of the initial AI prompt, the preference for concise output was dropped in favor of a chain-of-thought prompting technique. The hypothesis was that prompting the LLM to reason about drugs before reporting second-level ATC codes would lead to higher quality classifications as, at the time of making the classification, the LLM would have access to its own reasoning before outputting the ATC classes so that the LLM can use the text generated by the reasoning. Few-shot learning examples were used here as well, and the entire revised AI prompt is shown in Textbox 4 and made available as a plain text file in Multimedia Appendix 2.

As a side note, dexamethasone from the initial prompt examples was omitted from the revised prompt. It is a secondary classification of S01, an ophthalmological drug, would not be dispensed through spencer as it is a liquid eye drop.

The revised prompt was able to classify aspirin in the desired manner. For instance, in the OpenAI playground, the assistant responded to the input “aspirin|1 pill of 81mg” with the output presented in Textbox 5.

Despite the carriage returns, the output was pipe-delimited as requested, and the reasoning correctly specifies that aspirin that this dose is used for its antiplatelet effects rather than its pain relief effects. It did not include N02 as the next most likely drug classification, and this was deemed acceptable since a patient is unlikely to be taking 81 mg of aspirin daily for pain. An input of “aspirin|2 pills of 325mg” led to the output in Textbox 6.

Thus, the revised prompt with chain-of-thought reasoning returned N02 as the best ATC code for high-dose aspirin and B01 as the next-best second-level ATC. This was deemed acceptable as aspirin could conceivably be taken for a dual purpose, and the revised prompt was promoted to the pilot sample phase.

**Revised artificial intelligence prompt with chain-of-thought prompting techniques.**

#Instructions

You are a classifier of oral drug prescriptions into Anatomical Therapeutic Chemical (ATC) level 2 therapeutic subgroups. Handle different capitalizations, common misspellings, and concatenations with manufacturer names. As a drug and dose combination may be linked to several ATC level 2 subgroups, your job is to return the most likely and next most likely level 2 subgroups. The user’s input should take the from <Oral solid drug name>|<Daily drug dose>, whereby you should output a 3-column, pipe-delimited message taking the form: <Reasoning>|<Most likely level 2 ATC Code>|<Next most likely level 2 ATC Code (or “NA”)>. Reason step by step as demonstrated in the examples, taking into consideration the most likely indication given the daily dose.

#Examples

*Input: metformin|2 pills of 500MG

*Output: 2 pills of 500MG is 1000MG per day. The typical effective dose range for metformin in managing type 2 diabetes is between 500mg to 2000mg per d., and 1000MG is right within this range. The code A10 refers to “Drugs used in diabetes.”|A10|NA

*Input: apo-dexmethasone|0.5 pills of 4.0MG

*Output: 0.5 pills of 4.0 MG is 2.0 MG per day. Dexamethasone at this dose is typically used for mild to moderate inflammatory and autoimmune conditions. The primary code H02 refers to “Corticosteroids for systemic use, plain.”|H02|NA

*Input: XYZ-1234|1 pill of 1g

*Output: XYZ-1234 is not an oral solid drug I am familiar with.|NA|NA

*Input: webber naturals womens 50 plus most|1 pill of UNK

*Output: The dose is unintelligible, but this is a multivitamin and is classified by ATC Code A11: Vitamins.|A11|NA

**The output for aspirin at 81 mg per day.**

1 pill of 81mg is 81mg per day. Aspirin at this low dose is typically used for its antiplatelet effects to prevent cardiovascular events such as heart attacks and strokes. The primary ATC code for antithrombotic agents is B01.

Reasoning: Aspirin at 81mg is commonly used for cardiovascular protection rather than for pain relief or anti-inflammatory purposes, which would require higher doses.

|B01|NA

**The output of aspirin at 650mg per day.**

2 pills of 325mg is 650mg per day. Aspirin at this dose is typically used for pain relief, anti-inflammatory purposes, and sometimes for cardiovascular protection. The primary ATC code for aspirin when used for pain and inflammation is N02: “Analgesics.” When used for cardiovascular protection, the ATC code is B01: “Antithrombotic agents.” Given the dosage, it is more likely to be used for pain relief.

Reasoning: 2 pills of 325mg is 650mg per day. Aspirin at this dose is typically used for pain relief and anti-inflammatory purposes. The primary code N02 refers to “Analgesics.”

|N02|B01

### A Finite Population of Daily Drug Doses

To this point, all AI prompt testing was done via hypothetical examples. For testing on prescription drugs in a real population, 3371 deidentified patients met the inclusion criteria described in Textbox 1, of which 2908 (86.26%) resided in Canada and 463 (13.73%) resided in the United States. These patients collectively had 4.76 million doses scheduled after January 1, 2024, according to the database query date of June 1, 2024. Within these doses, there were 2077 distinct drug names, 517 (24.89%) of which were prescribed to patients residing in Canada, and 646 (31.1%) were prescribed to patients residing in the United States. Only 86 (4.14%) out of 2077 drug names were prescribed to patients residing in both regions. When combined with daily pill quantity and strength information, there were 12,294 daily drug prescription strings (eg, “pms-quetiapine|1 pill of 100.0 MG, 1 pill of 200.0 MG”), the finite population of interest.

### The Pilot Sample for Testing the AI Prompt

The random sample of 20 daily dosage strings was used to determine if the revised prompt would continue to be studied. The 20 daily dose strings appear in Table 2 alongside the output from the OpenAI Batch API.

This result was deemed adequate for proceeding, despite 2 issues. The string “apo-lamotrigine|0.5 pills of 25.0 MG” returned a correct best second-level ATC code of N03 but an incorrect next-best second-level ATC code of N05. For the string “apo-pregabalin|4 pills of 75.0 MG,” the first of several pregabalin doses to come, the antiepileptic nature is emphasized at the expense of the neuropathic pain aspect that the drug has come to be most associated with. The N02 best second-level ATC code would have arguably been superior.

**Table 2:** The pilot sample for testing the revised artificial intelligence (AI) prompt.

Input string for user role	Best second-level ATC^a^	Next-best second-level ATC
carbamazepin tab|2 pills of 200MG	N03	N/A^b^
calcium-ng-vitd - jam|1 pill of UNK	A12	N/A
teva-entacapone|7 pills of 200.0 MG	N04	N/A
apo-midodrine|8 pills of 2.5 MG	C01	N/A
hydroxyzine hydrochloride|4 pills of 10.0 MG	N05	R06
apo-domperidone|2 pills of 10.0 MG	A03	N/A
aventyl|4 pills of 25.0 MG	N06	N/A
sandoz perindopril erbumine/indapamide hd|2 pills of 0.0	C09	C03
vitamin d2|1 pill of 1.25 mg	A11	N/A
synthroid|2 pills of 125.0 MCG	H03	N/A
apo-lamotrigine|0.5 pills of 25.0 MG	N03	N05
jamp-azithromycin|2 pills of 250.0 MG	J01	N/A
metoprolol-l|3 pills of 50.0 MG	C07	N/A
sandoz irbesartan|1 pill of 75.0 MG	C09	N/A
apo-pregabalin|4 pills of 75.0 MG	N03	N06
apo-ramipril|1 pill of 10.0 MG, 1 pill of 5.0 MG	C09	N/A
odan bupropion sr|2 pills of 100.0 MG	N06	N07
valacyclovir tab|1 pill of 500MG	J05	N/A
olanzapine|3 pills of 10 MG	N05	N/A

^a^ATC: Anatomical Therapeutic Chemical Classification System.

^b^N/A: not applicable.

### The Inference Sample for Estimating Accuracy of AI Drug Classification

With the 20 records from the pilot sample excluded, a final sample of 200 daily drug dose strings were taken without replacement. These strings were sent to the OpenAI Batch API using GPT-4o with the settings previously described on July 10, 2024, for a total cost of US $0.33. Of the 200 daily dose string inputs, 175 (87.5%) were graded as perfect, 10 (5%) had a minor issue, and 15 (7.5%) had a major issue. For inference to our population of 12,294 daily drug prescription strings, the estimate of mostly correct outputs was 92.5% (185/200, 80% CI 90.1%-94.9%).

Despite not being tuned for an oral solid database, the pipeline based on Google’s Programmable Search Engine did well. Out of the 200 drug names submitted to the algorithm, 82.5% (165/200) were deemed acceptable for use in the oral solid database, while 17.5% (35/200) were not. Table 3 shows the breakout of grades from both algorithms.

**Table 3:** GPT-4o with prompt versus a pipeline using Google’s Programmable Search Engine and regular expressions.

Large language models score	Search-based algorithm score			Margin
	Acceptable	Not acceptable		
1 (perfect)	148	27	175	
2 (minor issue)	8	2	10	
3 (major issue)	9	6	15	
Margin	165	35	200	

All imperfect gradings from either algorithm are presented in Multimedia Appendix 3.

When discussing the imperfect grades from the LLM algorithm, pregabalin was the most frequent culprit, appearing total 7 times in the inference sample under different brand names and different dosing configurations. In each case, the LLM’s output returned N03 as the best second-level ATC code followed by N06. The search-based algorithm consistently returned N02, which aligns with pregabalin’s most well-recognized role of treating neuropathic pain. However, pregabalin, developed as an antiepileptic, is still used in that capacity and also for generalized anxiety disorder. Thus, the LLM's outputs were considered more correct than incorrect.

The largest category of serious errors is where the LLM either did not recognize a real drug or reasoned about the wrong drug. An example of the former was MYA, a Canadian birth control oral solid [41] that GPT-4o was unable to retrieve information about. Interactive prompt modifications in the OpenAI playground could not overcome this. In a second case, the Canadian morphine drug STATEX [42] was reasoned about as a statin, presumably due to the lexical similarity. However, this mistake could not be replicated within the OpenAI playground.

Reasoning about vitamins that might be anemia treatments caused two grade 3 errors relating to the best second-level ATC. The decision was made to require B03 (Antianemic preparations, a second-level ATC code) as the best second-level ATC code for Vitamin B12 supplements if the dose was high or if the dose was unknown, and in these 2 cases Vitamin B12 supplements were classified primarily as A11 (vitamins, a second-level ATC code). A11 was allowed as a next-best code if it was provided but was not required.

The vitamin D analog calcitriol proved to be another noteworthy case in the domain of vitamins. While Vitamin D analogs explicitly fall under A11, calcitriol is no ordinary vitamin, and its therapeutic use case falls better under H05 (calcium homeostasis). The decision was made to require both A11 and H05 for full credit, but to give mostly correct status if either one was present. In the 2 times that calcitriol appeared, only A11 was present in one case and only H05 in the other, so these were graded as more correct than incorrect.

The reasoning in the chain-of-thought responses was not without cost. The extra output costs money, and there’s additional likelihood of a delimiter error because the algorithm needs to stop reasoning and add the pipe delimiters and ATC codes. In total, 2 (1%) times out of the 200, delimiter errors were a primary cause of a mostly incorrect grade.

The LLM respected the oral solid criteria, sometimes to a fault. In a favorable case, azithromycin was classified as J01 when it would have been classified as S01 if packaged in liquid form as eye drops. However, the search-based algorithm also returned J01, without any oral solid prompting. For the case of a reminder pouch for Repatha, an injectable, the LLM was a reminder pouch for Repatha, an injectable, where the LLM only returned missing data points even though Repatha itself is easily classified as C10. This was not considered incorrect because the prompt was specifically asked to return NAs when drugs could not be classified as oral solids.

During the grading process, difficulties with the problem formulation of best versus next-best ATC code became apparent in ways that were not obvious from the motivating example of aspirin. Terazosin, which can be used as an antihypertensive or a urological in oral solid form and where doses overlap, was well served by a C02|G04 output. Calcium, magnesium, zinc, and Vitamin D3 were well served by an A12|A11 output. However, in other cases, things were less clear. Pregabalin could have at least 3 ATC codes. For the case of calcitriol, there are only 2 relevant ATC codes, but the order of best versus next best is not obvious.

The drug methotrexate illuminated a limitation of the daily dose formulation of the prompt. The string input was “pms-methotrexate|5 pills of 2.5 MG” which suggests a 12.5-mg daily dose; however, this medication is often dosed once per week. This output was graded as mostly incorrect as it returned L01 instead of L04, whereas the former would more typically be associated with high-dose infusions for cancer treatment.

After focusing the first part of this research on chain-of-thought reasoning about drug dose, it is natural to question whether this was worth the additional prompt complexity and number of prompts. While the reasoning would often mention “at this dose,” or mention that the dose fell within a common range of prescriptions, in the end, the results mostly aligned with the search-based algorithm. The cases where the dose mattered were antianemic preparations such as high-dose folic acid (a case that the search-based algorithm missed) and high-dose folate. It also helped to give prednisone an H02 systemic use classification as opposed to a higher dosage A07 classification for inflammatory bowel disease. Notably, low-dose aspirin did show up in the inferential sample, but the drug name was “aspirin low dose” and the search-based algorithm was easily able to reach a majority vote of B01.

## Discussion

### Principal Findings

LLMs such as GPT-4o perform well in the drug classification task. In the handpicked example of aspirin, GPT-4o was able to distinguish between 2 therapeutic uses based on the dose, which happened only after incorporating a chain-of-thought prompting technique. This prompt, when applied to a larger sample, was deemed mostly correct a vast majority of the time (n=200, 92.5%).

Google’s Programmable Search Engine via the Custom Search JSON API also does well with extracting ATC codes when combined with a simple pipeline using regular expressions and voting. While the proportion of appropriate search-algorithm responses (n=200, 82.5%) was somewhat lower than the mostly correct proportion mostly correct proportion from the LLM, the algorithm was not specifically tuned to the task of coding oral solids. Different search terms could be tried or more pages could be returned.

However, the question is not whether Google’s Programmable Search Engine could be tuned to outperform an LLM, because with enough time and effort, the answer can likely be found online. One place where such answers show up is a publicly accessible version of DrugBank, a proprietary source of drug information, that is indexed by Google’s Programmable Search Engine and often shows up in the summary snippets returned by the Custom Search JSON API. Along with Google’s restriction on persistent storage of results, automatic scraping of such data represents multiple terms-of-service violations. While the enforceability is questionable, why incur the risk when LLMs perform as well as they do and are meant to extract information for the user to keep for as long as is necessary?

In addition, Google Search, while quite reproducible in the short term due to caching, has faced allegations of declining quality [43] and its long-term reproducibility is uncertain. Prediction algorithms built upon Google’s Search infrastructure have failed when the infrastructure changed, as in the case of Google flu [44].

Reproducibility for versioned LLMs with temperature parameters like GPT-4o should, in principle, be perfect, as setting the temperature parameter to 0 should lead to deterministic output (at least when there are no probability ties between tokens). However, this is not true in practice [45]. Differences between the Batch API and OpenAI playground were sometimes material, and this has been experienced by other users. One user of the OpenAI Batch API commented that batching changed the behavior of an OpenAI LLM, making the results “too similar” [46]. At the time of writing, an experimental feature from OpenAI is available that includes a seed parameter and system fingerprint, which may be helpful in achieving perfect reproducibility.

Despite concerns with reliability in this research, GPT-4o’s reliability has been praised in medical contexts as higher than alternatives. In a study on extraction and summarization of Japanese-language clinical research protocols, GPT-4o was said to exhibit “high reproducibility,” with 80% and 100% accuracy for research objectives and research designs [47]. In an information extraction task on veterinary electronic health records, GPT-4o demonstrated greater reproducibility than human pairs, with an average Cohen κ of 0.98 versus 0.8 for humans [48].

For US $0.33, 200 daily drug prescription strings were classified into best and next-best second-level ATC codes with the reasoning provided. That would correspond to spending approximately US $20 to classify all 12,294 daily drug prescription strings in the SHS drug database. The affordability of GPT-4o as a drug categorization tool signifies a democratization of research instruments in pharmacoepidemiology, as the inexpensive use of GPT-4o, especially via the OpenAI Batch API, means that drug classification can be accommodated on virtually any research budget. Prompts can be shared within the research community, and the richness of drug datasets will be enhanced for teams worldwide. Furthermore, as competition increases, capabilities are likely to improve while prices fall. An example of this is GPT-4o itself, which at the time of release was twice as fast and half the price of GPT-4 Turbo [49]. This cost-effectiveness democratizes access to advanced drug classification tools and, as such, promotes equitable research opportunities.

### Limitations and Future Work

This study involved a specific population of daily drug prescriptions from polychronic patients in a value-based care organization residing in the United States and Canada. Geographically, the patients were biased toward Canada, with some US representation. Future work is needed to replicate drug classification in other settings. In addition, one of the motivations for One motivation for adopting LLMs was their ability to accommodate drugs in non-English speaking countries that use operational codes other than DIN and NDC. While GPT-4o boasts of “significant improvement on text in non-English languages” [49], such classification was out of scope for this research. Future studies are needed to test the global generalizability of this approach.

The manual grading used in this paper has the potential for bias and errors. The grading process was tedious and represented 220 separate research investigations. It is likely that contradictions remain; however, all imperfect grades were documented in the Results section. In the future, more formal methods, such as the Delphi method [50], could add additional rigor.

The problem formulation of best and next-best second-level ATC codes, based on daily dosage strings, was occasionally not ideal for the drugs and doses encountered. Examples include cases where more than 2 ATC codes were necessary, times where ordering was not clear, questionable relevance of a next-best code, and weekly dosing schedules. Other formulations could be considered, such as those that return true or false for a set of codes, potentially not based on dose at all. Ignoring the dose would greatly simplify the problem formulation. In addition to alleviating the daily versus weekly frequency complexity, the multiplicity of inputs is cut down to a fraction of what was encountered in this research, as only the drug name is the input.

Complete and total reproducibility of GPT-4o outputs was not possible at the time of writing. New features such as system fingerprints and seeds may address this and are an important topic for future research. One prompting technique to deal with a multiplicity of possible outputs is to sample them from the LLM with temperature set above 0 and use a voting process. This is the essence of the “self-consistency” prompting approach [46]. In addition, with powerful open-source LLMs becoming available, such as the Llama family of models [51], there is the question of whether full reproducibility is achievable when the researcher is running the model locally. If so, is there a reduction in the quality of the drug classification when using an open-source model that may pose a trade-off to reproducibility?

Outside of reproducibility, more research on the benefits of open-source LLMs for drug classification and related tasks is needed. For instance, open-source models run locally have the privacy advantage of not needing to have data leave any internal network but could open up other privacy risks. The economics of using an open-source LLM vs proprietary LLMs via APIs is also unclear. This research was limited to one LLM, the proprietary GPT-4o (specifically the version “gpt-4o-2024-05-13”).

New LLMs are arriving regularly, with new capabilities. Future research will be needed to evaluate the drug classification capabilities of the next generation of the next generation of models. Continuing assessment of the emergence of new AI models will enable the use of the most-effective and updated tools for pharmacoepidemiologic research.

### Conclusions

This research demonstrated that GPT-4o is a powerful and accessible tool for enhancing pharmacoepidemiologic research by automating drug classification. GPT-4o and LLMs in general represent an inexpensive and straightforward method for augmenting real-world drug databases with Anatomical Therapeutic Chemical drug classes. This gives nearly all research teams access to a powerful tool to satisfy a key prerequisite of pharmacoepidemiological analysis using data from electronic health records, pharmacy management systems, and claims records. It is not just a matter of increased efficiency but also democratizing access to high-quality pharmacoepidemiologic drug classification instruments. Better, more accessible drug information is a precursor to higher quality and greater quantity of pharmacoepidemiologic datasets and a path toward better drug prescription policy and clinical outcomes for patients across the world.

## Appendix

Multimedia Appendix 1 The initial artificial intelligence (AI) prompt: few-shot learning with concise output.

Multimedia Appendix 2 Revised artificial intelligence (AI) prompt with chain-of-thought prompting techniques.

Multimedia Appendix 3 Imperfect classifications made by either the large language model (LLM)–based algorithm or Google’s search-based algorithm.

## Abbreviations

A11: vitamins (a second-level ATC code)
AI: artificial intelligence
API: application programming interface
ATC: Anatomical Therapeutic Chemical Classification System
B01: antithrombotic agents (a second-level ATC code)
DIN: Drug Identification Number
JSON: JavaScript Object Notation
LLM: large language model
N02: analgesics (a second-level ATC code)
NDC: National Drug Code
RWD: real-world data
SHS: Spencer Health Solutions

## Data Availability

A subset of the data generated during this study—specifically, examples of large language model–generated errors and corresponding processed drug and dose strings—is included in the supplementary information files. The complete set of processed drug strings and evaluation outcomes is available from the corresponding author on reasonable request. The raw data containing numerical identifiers are not publicly available due to privacy and confidentiality restrictions.
