# Supplementary material for: AI-Powered Drug Classification and Indication Mapping for Pharmacoepidemiologic Studies: Prompt Development and Validation
Source: JMIR AI. 2025 Jun 12;4:e65481. doi: 10.2196/65481 (PMC12203024; doi:10.2196/65481)
Supplement: Multimedia Appendix 3 [file ai_v4i1e65481_app3.docx]

Appendix. Imperfect classifications made by either the large language model (LLM)–based algorithm or the Google’s search-based algorithm.

| ID | LLM input string | Search input string | LLM best second-level ATC^a^ code | LLM next-best second-level ATC code | Custom Search API^b^ extracted codes | LLM score | Custom Search outcome | Comments |
| --- | --- | --- | --- | --- | --- | --- | --- | --- |
| 25 | sandoz folic acid\|1 pill of 5.0 MG | sandoz folic acid ATC code | B03 | N/A^c^ | {‘V03’: 2, ‘B03’: 1, ‘N02’: 1, ‘N01’: 1, ‘L01’: 1, ‘N05’: 1} | 1 | Not acceptable | This is a therapeutic dose of folic acid for treating anemia, so B03 is correct. V03 is incorrect as returned by the search algorithm |
| 34 | kadian sr\|1 pill of 10.0 MG | Kadian sr ATC code | N02 | N/A | {‘N02'’: 2, ‘A07’: 2} | 1 | Not acceptable | Regarding A07 returned by the search algorithm, morphine is a treatment for severe diarrhea, but is not relevant for this drug database |
| 36 | jamp-mycophenolate\|4 pills of 500.0 MG | jamp-mycophenolate ATC code | L04 | N/A | {‘J01’: 2, ‘L04’: 1, ‘S01’: 1, ‘R03’: 1, ‘L01’: 1, ‘N06’: 1} | 1 | Not acceptable | The search algorithm produced J01, which is incorrect |
| 37 | reminder for probiotics\|1 pill of UNK^e^ | reminder for probiotics ATC code | A07 | N/A | {‘A02’: 1} | 2 | Not acceptable | The LLM algorithm should have a next-best ATC code for probiotics, as there are many to choose from. A02 is not relevant for probiotics |
| 38 | calcium mag zinc+vit d3 \|1 pill of UNK | calcium mag zinc+vit d3 ATC code | A12 | A11 | {‘M05’: 1, ‘A11’: 1} | 1 | Not acceptable | Search algorithm failed to reach a consensus, does not include A12 |
| 46 | allopurinol\|4 pills of 100MG | allopurinol ATC code | M04 | N/A | {‘'C02’: 2, ‘R01’: 1, ‘G01’: 1, ‘N06’: 1} | 1 | Not acceptable | The search algorithm does not include M04 |
| 47 | prednisone\|2 pills of 1 mg | Prednisone ATC code | H02 | N/A | {‘H02’: 3, ‘S02’: 1, ‘A10’: 1, ‘A07’: 1} | 2 | Acceptable | LLM output should have included A07 as the next-best ATC |
| 54 | xylac\|4 pills of 10.0 MG, 2 pills of 25.0 MG | xylac ATC code | R06 | N/A | {‘N05’: 4} | 3 | Acceptable | The LLM algorithm incorrectly classified xylac as a treatment for allergies rather than schizophrenia |
| 57 | hydrochlorothiazide\|0.5 pills of 25.0 MG | Hydrochlorothiazide ATC Code | C03 | N/A | {‘C03’: 3, ‘C09’: 3} | 1 | Not acceptable | The search algorithm failed to reach a consensus. C09 is a combination product with a diuretic |
| 59 | jamp-vitamin d\|1 pill of 50000.0 UNIT | jamp-vitamin d ATC code | A11 | N/A | {‘'M05’: 1, ‘A11’: 1} | 1 | Not acceptable | For the search algorithm, vitamin D fails to get an A11 majority |
| 60 | jam-chewable vitamin c\|1 pill of UNK | jam-chewable vitamin c ATC code | A11 | N/A | {‘A11’: 1, ‘D03’: 1, ‘G04’: 1} | 1 | Not acceptable | For the search algorithm, vitamin C fails to get an A11 majority |
| 64 | pms-terazosin\|1 pill of 2.0 MG | pms-terazosin ATC code | C02 | G04 | {‘G04’: 2, ‘N04’: 2} | 1 | Not acceptable | The search algorithm was unable to arrive at a consensus |
| 68 | pms-pregabalin\|2 pills of 25.0 MG | pms-pregabalin | N03 | N06 | {‘N02’: 4} | 2 | Acceptable | The case of pregabalin |
| 72 | apo-naltrexone\|1.5 pills of 50.0 MG | apo-naltrexone ATC code | N07 | N/A | {‘N02’: 3, ‘N07’: 1} | 1 | Not acceptable | N02 is a combination product |
| 73 | reminder for repatha\|1 pill of UNK | reminder for repatha ATC code | N/A | N/A | {‘C10’: 1, ‘A02’: 1} | 1 | Not acceptable | The search algorithm could not get a majority. The LLM output indicates correctly that this is not an oral solid; however, something is lost by not classifying the drug |
| 74 | jamp famotidine\|1 pill of 40.0 MG | jamp famotidine ATC code | A02 | N/A | {‘A02’: 1, ‘L04’: 1, ‘J05’: 1} | 1 | Not acceptable | Search-based algorithm is unable to obtain a majority vote |
| 76 | novo−ferrogluc\|1 pill of 300.0 MG | novo-ferrogluc ATC code | B03 | N/A | {‘B03’: 1} | 1 | Acceptable | B03 is a good choice here, given that this is an iron supplement, and the dose is in a range for treating anemia |
| 84 | turmeric (qunol) capsule\|1 pill of 1500.0 mg | turmeric (qunol) capsule ATC code | A16 | —^e^ | {} | 3 | Acceptable | The LLM incorrectly returned ATC codes for a dietary supplement. There was also a delimiter error, which resulted in the description’s choice, M01, not being displayed, and the second most likely code being displayed in the top position |
| 87 | sandoz sodium bicarbonate\|4 pills of 500.0 MG | sandoz sodium bicarbonate ATC code | A02 | N/A | {} | 1 | Not acceptable | Search algorithm failed to return results |
| 89 | compounded naltrexone capsule\|3 pills of 0.25 mg | compounded naltrexone capsule ATC code | N02 | — | {‘N02’: 2, ‘N07’: 1}) | 3 | Not acceptable | The description within the LLM says that it is N07, but there was a delimiter error |
| 93 | euro−fer\|1 pill of UNK | euro-fer ATC code | B03 | N/A | {‘J07’: 1, ‘M05’: 1} | 1 | 3 | The drug is marketed as an antianemia preparation |
| 95 | apo-pregabalin\|2 pills of 75.0 MG | apo-pregabalin ATC code | N03 | N06 | {‘N02’: 3, ‘N03’: 1} | 2 | Not acceptable | The case of pregabalin |
| 96 | turmeric curcumin x-strength\|2 pills of UNK | turmeric curcumin x-strength ATC code | A16 | N/A | {‘L01’: 1} | 3 | Not acceptable | Dietary supplement classified as drug |
| 97 | keto 600 exogenous\|2 pills of UNK | keto 600 exogenous ATC code | A16 | N/A | {‘L02’: 1, ‘A14’: 1, ‘G03’: 1 | 3 | Not acceptable | Dietary supplement classified as drug |
| 99 | vitamin b12 sl\|2.5 pills of UNK | vitamin b12 sl ATC code | A11 | N/A | {‘B03’: 2} | 3 | Acceptable | LLM did not include B03, for antianemia preparations, which, to be consistent, is required for vitamin B12, regardless of the dose |
| 100 | align\|1 pill of 4.0 MG | Align ATC code | N06 | N/A | {} | 3 | Acceptable | The LLM description treats a probiotic as an Alzheimer drug |
| 112 | mar-diltiazem t\|1 pill of 240.0 MG | mar-diltiazem t ATC code | C08 | N/A | {‘C08’: 1, ‘B01’: 1} | 1 | Not acceptable | No majority in search algorithm results |
| 115 | naltrexone hcl\|0.5 pills of 50.0 MG | naltrexone hcl ATC code | N07 | N/A | {‘V03’: 3, ‘N07’: 2, ‘N02’: 1} | 1 | Not acceptable | V03 is not the correct code |
| 119 | calcitriol-odan\|1 pill of 0.5 MCG | calcitriol-odan ATC code | A11 | N/A | {‘D05’: 2, ‘A11’: 2, ‘A06’: 1} | 2 | Not acceptable | A decision was made to require both A11 and H05 for the LLM output. D05 is correct for topical formulation but is not ideal for an oral solid database |
| 120 | jamp-pregabalin\|2 pills of 50.0 MG | jamp-pregabalin | N03 | N06 | {‘N02’: 4, ‘N03’: 1} | 2 | Acceptable | The case of pregabalin |
| 126 | xylac\|0.5 pills of 10.0 MG | xylac ATC code | R06 | N/A | {‘N05’: 3} | 3 | Acceptable | Xylac treats psychosis, not allergies |
| 133 | one-a-day womens 50+ advantage\|1 pill of 0.0 | one-a-day womens 50+ advantage ATC code | A11 | N/A | {} | 1 | Not acceptable | No result from the search-based algorithm |
| 140 | sandoz clonidine\|6 pills of 0.025 MG | sandoz clonidine ATC code | C02 | N/A | {‘S01’: 3, ‘N02’: 2, ‘C02’: 1, ‘N06’: 1} | 1 | Not acceptable | S01 is relevant for eye drops, so not wrong, but not ideal for an oral solid database |
| 141 | jamp-magnesium\|1 pill of 100.0 MG | jamp-magnesium ATC code | A12 | N/A | {‘A02’: 2, ‘C03’: 1, ‘G04’: 1, ‘A04’: 1, ‘A12’: 1} | 1 | Not acceptable | The search algorithm comes back with false positives |
| 147 | sandoz colchicine\|1 pill of 0.6 MG | sandoz colchicine ATC code | M04 | N/A | {‘N02’: 1, ‘J01’: 1, ‘R03’: 1, ‘M01’: 1, ‘M04’: 1, ‘C10’: 1} | 1 | Not acceptable | The search algorithm comes back with false positives |
| 152 | sodium chloride\|2 pills of UNK | sodium chloride ATC code | A12 | N/A | {‘B05’: 3, ‘A12’: 1, ‘R01’: 1} | 1 | Not acceptable | B05 would relate to infusions, which is not an ideal code for an oral solid database |
| 157 | pregabalin\|6 pills of 25.0 MG | Pregabalin ATC code | N03 | N06 | {‘N02’: 6, ‘N03’: 1} | 2 | Acceptable | The case of pregabalin |
| 158 | d3-50\|1 pill of 50000.0 UNIT | d3-50 ATC code | A11 | N/A | {‘M05’: 2, ‘A11’: 2} | 1 | Not acceptable | D3 is an oral solid, and M05 implies infusions |
| 159 | calcitriol\|1 pill of .25 ug | Calcitriol ATC code | H05 | N/A | {‘A11’: 5, ‘D05’: 3, ‘A06’: 1} | 2 | Acceptable | A decision was made to require both A11 and H05 for the LLM output. A11 is accepted from the search-based algorithm |
| 161 | l-methylfolate calcium\|1 pill of 7.5 MG | l-methylfolate calcium ATC code | B03 | N/A | {‘V03’: 3, ‘B03’: 1, ‘D11’: 1, ‘N02’: 1} | 2 | Not acceptable | B03 is correct as this is a form of folate and clearly in the therapeutic dose range |
| 162 | reminder for vitamin b12\|1 pill of UNK | reminder for vitamin b12 ATC code | B03 | N/A | {‘M05’: 1 | 1 | Not acceptable | A decision was made to accept B03 for vitamin B12, regardless of the dose |
| 169 | apo-pregabalin\|2 pills of 150.0 MG, 4 pills of 50.0 MG | apo-pregabalin ATC code | N03 | N06 | {‘N04’: 7} | 2 | Acceptable | The case of pregabalin |
| 172 | mya\|1 pill of 0.0 | mya ATC code | N/A | N/A | {} | 3 | Not acceptable | MYA^f^ is a Canadian birth control pill |
| 178 | concerta\|2 pills of 54.0 MG | concerta N07 | N07 | — | {‘N06’: 7} | 3 | Acceptable | There was a delimiter error made in the LLM algorithm. The description does have N06 as the best code |
| 179 | pms-pregabalin\|2 pills of 150.0 MG | pms-pregabalin ATC code | N03 | N06 | {‘N02’: 4} | 2 | Acceptable | The case of pregabalin |
| 187 | apo-lithium carbonate\|3 pills of 150.0 MG | apo-lithium carbonate ATC code | N05 | N/A | {‘N02’: 2, ‘C01’: 1, ‘N05’: 1} | 1 | Not acceptable | A clear case of treatment for bipolar |
| 191 | lyrica\|8 pills of 75.0 MG | Lyrica ATC code | N03 | N06 | {‘N02’: 6, ‘N03’: 2} | 2 | Acceptable | The case of pregabalin |
| 194 | webber omega 3-6-9\|1 pill of 1200.0 mg | webber omega 3-6-9 ATC code | A11 | N/A | {} | 3 | Not acceptable | The best code should have been C10 |
| 195 | statex\|4 pills of 5.0 MG | statex ATC code | C10 | N/A | {‘N02’: 1} | 3 | Acceptable | This is a brand name for morphine |
| 201 | b12\|1 pill of UNK | b12 ATC code | A11 | N/A | {‘B03’: 4} | 1 | Acceptable | The decision was made to require B03 for vitamin B12 regardless of the dose |
| 205 | pms-methotrexate\|5 pills of 2.5 MG | pms-methotrexate ATC code | L01 | M01 | {‘L04’: 5, ‘L01’: 1} | 3 | Acceptable | L04 would be the correct best ATC code for an oral solid, although it is important to note that methotrexate is often given in a single weekly dosage, so this shows a limitation of the prompt formulation |
| 206 | hanzema\|1 pill of 30.0 MG | hanzema ATC code | A02 | N/A | {‘D05’: 2, ‘D11’: 2, ‘L01’: 2} | 3 | Not acceptable | The correct answer was D11, as hanzema is indicated for the treatment of severe chronic hand eczema. The search-based algorithm failed to reach a majority |
| 216 | vitamin c timed release\|2 pills of UNK | vitamin c timed release ATC code | A11 | N/A | {‘A11’: 1, ‘N06’: 1, ‘M03’: 1} | 1 | Not acceptable | Search-based algorithm failed to reach a majority |
| 220 | calcium magnesium with zinc\|3 pills of 0.0 | calcium magnesium with zinc ATC code | A12 | N/A | {‘B05’: 2, ‘A12’: 2, ‘A02’: 2} | 1 | Not acceptable | Search-based algorithm failed to reach a majority |

^a^ATC: Anatomical Therapeutic Chemical Classification System.

^b^API: application programming interface.

^c^N/A: not applicable.

^d^UNK: “Unknown,” a result of a dose that was not able to be casted to a number during preprocessing.

^e^Not available.

^f^MYA: A birth control drug marketed in Canada by APOTEX INC.
